# Rapid frontotemporal gray matter loss in proposed body-first Parkinson’s disease: a longitudinal voxel-based morphometry study

**DOI:** 10.3389/fneur.2025.1579561

**Published:** 2025-07-23

**Authors:** Jin Yan, Ge Yao

**Affiliations:** ^1^Department of Radiology, Xinhua Hospital Affiliated to Shanghai Jiao Tong University School of Medicine, Shanghai, China; ^2^Department of Neurology, Xinhua Hospital Affiliated to Shanghai Jiao Tong University School of Medicine, Shanghai, China

**Keywords:** MRI, Parkinson’s disease, REM sleep behavior disorder, cerebrospinal fluid, dopamine transporter asymmetry

## Abstract

**Objective:**

The study aimed to compare the progressive gray matter (GM) atrophy between brain-first and body-first Parkinson’s disease (PD) under assumption.

**Methods:**

Based on the hypothesis that the timing of rapid eye movement (REM) sleep behavior disorder (RBD) onset relative to motor symptoms may differentiate brain-first from body-first PD subtypes, we stratified PD patients from the Parkinson’s Progression Markers Initiative (PPMI) database into 28 RBD-positive PD patients (PDRBD+), 26 RBD-negative PD patients (PDRBD-), 33 isolated RBD (iRBD) patients, and 35 healthy controls. PDRBD+ and PDRBD− groups underwent 4 visits within 48 months including clinical assessments and structure MRI. We measured GM atrophy cross-sectionally and longitudinally and analyzed their associations with other proposed markers.

**Results:**

At baseline, all groups were comparable and showed no significant difference in GM volume (GMV). The longitudinal GMV analysis (group-by-time interaction) showed that, compared with PDRBD− group, PDRBD+ demonstrated significant GM loss in the medial surface of the frontal lobes and the left temporal lobe. In the comparison at the 48th month, besides the bilateral frontotemporal lobes, the PDRBD+ group showed significant GM atrophy in the bilateral cerebellum. The two groups had no significant differences in the 12th and 24th months. Over time, each PD group showed extensive cortical GM loss and bilateral caudate and putamen atrophy. The altered GMV (interaction effect) was positively associated with the MoCA scores and negatively with SCOPA-AUT. Furthermore, the group-level F-statistic map highlighted spatially distinct differences in the protein metabolism, signal transduction, and autophagy pathways between PDRBD+ and PDRBD− groups.

**Conclusion:**

Body-first PD patients show a relatively rapid GMV loss in specific frontotemporal regions (e.g., left middle temporal gyrus) within 6 years following motor symptoms. Our findings add further evidence to the *α*-synuclein spreading hypothesis.

## Introduction

Parkinson’s disease (PD) is characterized by the existence of *α*-synuclein (α-syn) within neurons, known as Lewy pathology. Growing evidence indicates that abnormally folded *α*-syn protein exhibits prion-like behavior, causing the spread of pathology from one cell to another (1, 2). The progression of *α*-syn pathology is often described as moving from the lower brainstem to the upper regions, affecting the dorsal motor nucleus, the midbrain, and limbic structures sequentially following the six Braak stages defined by neuropathological research (3).

Recent evidence has indicated that the current staging scheme may not sufficiently capture the individual differences in PD’s pathophysiological development (4). Some researchers have hypothesized two distinct subtypes of *α*-syn propagation on the basis of the anatomical origin of α-syn and its subsequent spread patterns. These subtypes, termed the brain-first and body-first subtypes, offer contrasting pathways of pathology dissemination. The brain-first subtype, also named the top-down subtype, refers to an *α*-syn pathology pathway starting from the brain to the peripheral autonomic nervous system via the olfactory bulb (5–7). In contrast, the body-first subtype, also named the bottom-up subtype, refers to a pathway from the peripheral autonomic nervous system to the central nervous system via the vagus nerve and sympathetic connectome. This hypothesis posits that premotor rapid eye movement (REM) sleep behavior disorder (RBD) before parkinsonism is a robust indicator of the body-first subtype (8). Clinical and imaging evidence suggests body-first PD patients display more peripheral tissue pathologies, symmetric striatal dopaminergic losses, and autonomic and motor symptoms. On the contrary, body-first PD patients only present minor changes in cortical metabolism prior to motor symptoms (9).

Following the concept described earlier, both faster cortical involvement and absence of RBD would be suitable proxies for the brain-first PD subtype (10). Based on the hypothesis that RBD status may reflect distinct PD pathological subtypes (8), we conducted the current study to compare the gray matter volume (GMV), a neurodegeneration marker, between *de novo* PD subjects with (PDRBD+) and without RBD (PDRBD-) at baseline. Furthermore, isolated RBD (iRBD) represents a recognized prodromal stage of body-first PD. Its inclusion validates shared clinical and GMV features with body-first PD, and their collective divergence from the brain-first PD subtype. While a current case–control study did not succeed in distinguishing the patterns of brain atrophy between the two subtypes (11), this differentiation may become apparent during long-term follow-up. Therefore, we first performed a cross-sectional analysis to detect GMV differences among PDRBD+, PDRBD-, iRBD, and healthy controls (HCs). Then, we conducted a longitudinal analysis to identify the different patterns of gray matter (GM) atrophy between PDRBD+ and PDRBD-. Finally, we examined the correlations between GMV and other clinical variables using correlation analysis.

## Subjects and methods

### Participants

We used data from the Parkinson’s Progression Markers Initiative (PPMI) database,^1^ which implements standardized protocols as detailed in their official documentation.^2^ Ethical approval was granted by the institutional review board, and written informed consent was obtained from all participants. This study included 28 RBD-positive PD patients (PDRBD+), 26 RBD-negative PD patients (PDRBD-), 33 iRBD patients, and 35 HCs. Based on the hypothesis that RBD status may reflect pathological subtypes, PDRBD+ was used as a proxy for body-first PD while PDRBD− was considered potentially representative of brain-first PD, recognizing that this classification has limitations. PD patients were included if they had magnetic resonance imaging (MRI) scans available at baseline, 12-, 24-, and 48-month visits. Given limited longitudinal MRI data availability, HCs and iRBD patients were included with baseline MRI scans and matched to PD patients for age and sex distribution. Their IDs are shown in Supplementary material.

### Clinical, CSF *α*-syn, and dopamine transporter asymmetry evaluations

While polysomnography (PSG) remains the gold standard, RBD was assessed by using the REM Sleep Behavior Disorder Screening Questionnaire (RBDSQ) as a pragmatic alternative given the very restricted PSG data availability. The RBDSQ is a 10-item patient self-rating questionnaire covering the clinical features of RBD, such as dream enactment behaviors and related motor manifestations during sleep. Each item is scored dichotomously (0 = “no” or 1 = “yes”), with a total score range of 0–13. RBDSQ has been validated with sound diagnostic performance in the general population (12). Subjects scoring 3–4 were excluded to minimize classification ambiguity near the initially proposed cutoff of 5 (13), as these intermediate scores might represent a transitional or ambiguous group. Therefore, scores ≥ 5 indicate the presence of RBD (*n* = 28), and scores ≤ 2 indicate the absence of RBD (*n* = 26).

Motor symptoms were assessed by part III of the Movement Disorder Society Unified Parkinson’s Disease Rating Scale (MDS-UPDRS III) and Hoehn and Yahr (H-Y) staging without drug use. Cognitive performance was evaluated using the Montreal Cognitive Assessment (MoCA) scale, while the autonomic symptoms were assessed by the scales for outcomes in Parkinson’s disease-autonomic (SCOPA-AUT).

The participants’ cerebrospinal fluid specimens (CSF) were collected by lumbar puncture within a month of the MRI scan at baseline. A promising biomarker test, the seeding aggregation assay (SAA), was used to detect the pathological aggregation-inducing *α*-syn in the CSF. Meanwhile, CSF α-syn levels were analyzed using a commercially available enzyme-linked immunosorbent assay kit. Details about CSF proteins can be found in the PPMI biologics manual.^3^

We computed an asymmetry index (AI) of striatal binding ratios (SBR) to measure the asymmetry of dopaminergic degeneration based on the putaminal DaT SPECT and PPMI’s formula: Putamen AI = |100 × [(SBRleft − SBRright)/(mean (SBRleft + SBRright))]|.

### MRI study

Siemens 3.0 T scanners were used for all 3D T1-weighted images with the following scanning parameters: repetition time (TR) at 2,300 ms, echo time (TE) at 2.98 ms, inversion time at 900 ms, flip angle at 90°, slice number at 176, acquisition matrix of 240 × 256, and voxel size of 1 × 1 × 1 mm^3^.

Voxel-based morphometry (VBM) analysis is a widely used neuroimaging technique to investigate brain structural changes. In this study, we performed VBM using the Computational Anatomy Toolbox (CAT)12 Toolbox^4^ within the Statistical Parametric Mapping (SPM12) software.^5^

The longitudinal data processing batch provided by the CAT12 Toolbox was used to process the imaging data. The steps involved in the VBM analysis were as follows.

First, bias-corrected and inverse-consistent realignment was applied to calculate the realigned image means among all the time points. Next, spatially normalized values were estimated based on the segmentations of the mean image, using Diffeomorphic Anatomical Registration Through Exponential Lie algebra normalization, which can segment and modulate the images from all time points. The images were segmented into GM, white matter, and CSF components and modulated to ensure relative GMV. Afterward, we displayed one slice for each image to assess the spatial registration quality. Image quality was assessed by the mean correlation, Mahalanobis distance, total intracranial volume (TIV), and weighted overall image quality. We excluded images with artifacts, with abnormally enlarged ventricles, or without brain tissues. Finally, the images were smoothed using a 6 mm full-width-half-maximum isotropic Gaussian kernel. This step reduced image noise and improved spatial resolution.

### Transcriptomic analysis

We integrated transcriptome data to offer a potential mechanistic basis for structural measurements. The transcriptional datasets, containing gene expression information, were obtained from the Allen Human Brain Atlas (AHBA). Using a recently developed rigorous preprocessing pipeline (14), we ultimately identified 15,633 unique genes. We then examined the correlation between the expression profiles of each of the 15,633 genes from the AHBA and the group-level F-statistic map from the longitudinal study. To conduct the gene-set analysis, we utilized the online tool Metascape,^6^ using the top genes as input.

### Statistical analysis

Group comparisons of continuous variables were conducted by the two-sample independent t-test, or multiple-sample one-way analysis of variance (ANOVA) followed by Tukey’s *post hoc* tests. Group comparisons of categorical variables were conducted by χ2 tests. Longitudinal changes in clinical data over time were assessed using a linear mixed-effects model. Statistical significance was defined as *p* < 0.05 by two-tail tests. All data were analyzed using IBM SPSS Statistics 25.0 software.

One-way analysis of covariance (ANCOVA) was conducted to compare the GMV among four groups at baseline and between two groups at follow-up separately. Multivoxel comparisons were conducted to calculate the False discovery rate (FDR) while controlling for age, education years, and TIV.

In the longitudinal study, a flexible-factorial model was used to determine the GMV group-by-time interaction with two groups and four time points in the CAT12 toolbox, followed by a *post hoc* test to identify the GMV alteration direction (FDR-corrected *p* < 0.05).

The total brain region that was significantly different during the two-group comparison was used as a mask to extract each subject’s total voxel value. Pearson’s correlation was conducted to assess the correlations between the significant total voxel value and UPDRS-III, MoCA scores, SCOPA-AUT, and putamen AI at 48th month in the PDRBD+ and the PDRBD-, respectively, with *p* < 0.05 indicating statistical significance.

## Results

### Baseline characteristics and GMV analysis

According to the baseline RBDSQ, PD patients were divided into PDRBD+ and PDRBD-. Groups. Table 1 showed their baseline demographic and clinical characteristics. Age and gender were comparable among the PDRBD+, PDRBD-, iRBD, and HCs groups, with no significant differences. The HCs group had higher education and MoCA scores than all other groups. Disease duration, H-Y staging, UPDRS-III, and MoCA scores were comparable between the PDRBD+ and PDRBD− groups, with no significant difference. Compared to the HCs group, both PDRBD+ and iRBD groups showed greater autonomic dysfunction. CSF *α*-syn showed no difference among the groups. CSF SAA and putamen AI can effectively distinguish between the PD and HCs groups but not between the PDRBD+ and PDRBD− groups. None of the enrolled patients had pathological Leucine-Rich Repeat Kinase 2 (LRRK2), Glucosylceramidase Beta (GBA), or Alpha-synuclein (SNCA) variants. No cluster survived at FDR-corrected *p* < 0.05 at the baseline GMV comparison among groups.

**Table 1:** Demographics and clinical characteristics of PD patients, iRBD patients and HCs at baseline.

Clinical variables	PDRBD+	PDRBD−	iRBD	HCs	^a^ *p*	PDRBD+ vs. PDRBD−	PDRBD+ vs. HCs	PDRBD− vs. HCs	iRBD vs. HCs
Sample size	28	26	33	35					
Age (years)	63.87 ± 9.98	61.98 ± 8.84	64.91 ± 3.79	61.77 ± 10.13	0.395	0.847	0.767	1.000	0.432
Gender (F/M)	7/21	6/20	7/26	10/25	0.911	0.561	0.489	0.428	0.338
Education (years)	15.17 ± 2.73	14.27 ± 2.66	15.39 ± 3.16	17.17 ± 2.51	**0.001**	0.628	**0.028**	**0.001**	**0.047**
symptoms duration (months)	22.75 ± 16.99	27.07 ± 45.59	-	-	-	0.641^b^	-	-	-
H & Y	2 (1,2)	2 (1,2)	-	-	**-**	0.496^b^	**-**	**-**	**-**
MDS-UPDRS-III	24.96 ± 10.49	21.08 ± 7.67	-	-	-	0.129^b^	-	-	-
SCOPA-AUT	17.35 ± 13.64	10.73 ± 8.33	25.67 ± 14.46	9.48 ± 7.79	**<0.001**	0.152	**0.038**	0.975	**<0.001**
MoCA	26.78 ± 2.48	27.42 ± 2.02	26.88 ± 2.49	28.14 ± 1.11	**0.035**	0.675	0.054	0.542	0.064
Putamen AI	41.68 ± 25.87	31.10 ± 22.01	-	10.41 ± 6.73	**<0.001**	0.121	**<0.001**	**<0.001**	**-**
CSF α-syn (pg/mL)	1432.32 ± 639.01	1493.96 ± 688.69	-	1,590 ± 607.90	0.626	0.935	0.608	0.833	**-**
CSF SAA (P/N)	26/1	26/0	-	1/33	**<0.001**	0.509	**<0.001**	**<0.001**	**-**

PD, Parkinson’s disease; RBD, REM sleep behavior disorder; PDRBD+, RBD-positive PD patients; PDRBD−, RBD-negative PD patients; iRBD, isolated RBD; HCs, healthy controls; F/M, female/male; H & Y, Hoehn and Yahr staging; MDS-UPDRS III, part III of the Movement Disorder Society Unified Parkinson’s Disease Rating Scale; SCOPA-AUT, scales for outcomes in Parkinson’s disease-autonomic; MoCA, Montreal Cognitive Assessment; AI, asymmetry index; CSF, cerebrospinal fluid; SAA, seeding aggregation assay; P/N, positive/negative. ^a^*p* value refer to ANOVA models or chi-square test among groups. Post hoc tests were performed with Tukey’s correction. ^b^*p* value refer to independent t test or chi-square test between PDRBD+ and PDRBD− groups. Bold font indicates significant differences (*p* < 0.05).

### Longitudinal clinical and DaT asymmetry changes

**Figure 1:**
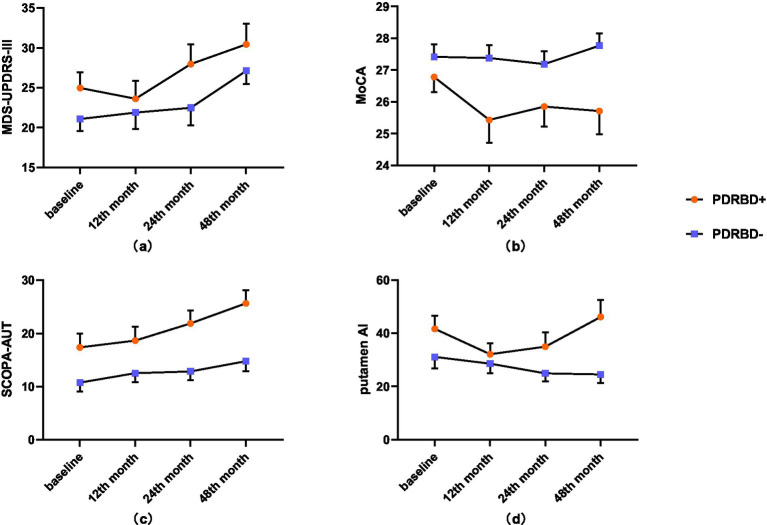
Longitudinal changes in clinical and imaging measures between the PDRBD+ and PDRBD− groups. **(a)** MDS-UPDRS-III, **(b)** MoCA, **(c)** SCOPA-AUT, and **(d)** putamen AI. Error bars represent standard errors of the mean. PD, Parkinson’s disease; RBD, REM sleep behavior disorder; PDRBD+, RBD-positive PD patients; PDRBD−, RBD-negative PD patients; MDS-UPDRS III, part III of the Movement Disorder Society Unified Parkinson’s Disease Rating Scale; SCOPA-AUT, scales for outcomes in Parkinson’s disease-autonomic; MoCA, Montreal Cognitive Assessment; AI, asymmetry index.

Table 2, Supplementary Table 1, and Figure 1 showed the clinical and DaT asymmetry changes during the follow-up. No group-by-time interaction was found in UPDRS-III, MoCA scores, SCOPA-AUT, or putamen AI. PDRBD+ group exhibited more severe cognitive impairment (*p* = 0.034) and automatic disorders (*p* = 0.034) than PDRBD−. Both groups had comparable MDS-UPDRSIII and putamen AI without significant differences. Over time, each PD group showed progressive aggravation in UPDRS-III and SCOPA-AUT (Supplementary Table 2). In the 48th month, the PDRBD+ group showed significantly increased putamen AI than PDRBD−.

**Table 2:** Summary statistics and results from mixed model analysis of change in outcomes from baseline, by visit.

Clinical variables	Mean change from baseline (SD)		Mixed model analysis	
	PDRBD+	PDRBD−	Difference (95% CI)	*p* value
MDS-UPDRSIII				
12th month	−1.36 (2.13)	0.83 (1.49)	−0.49 (−4.61 to 3.62)	0.812
24th month	3.00 (2.13)	1.57 (1.49)		
48th month	5.46 (2.13)	6.04 (1.49)		
MoCA				
12th month	−1.36 (0.59)	−0.38 (0.43)	1.49 (0.12 to 2.87)	0.034
24th month	−0.93 (0.59)	−0.23 (0.43)		
48th month	−1.07 (0.59)	0.35 (0.43)		
SCOPA-AUT				
12th month	1.29 (1.74)	1.97 (1.10)	−3.55 (−6.80 to −0.28)	0.034
24th month	4.50 (1.74)	1.95 (1.10)		
48th month	8.29 (1.74)	4.03 (1.10)		
Putamen AI				
12th month	−9.53 (6.14)	−2.42 (4.82)	−7.46 (−15.82 to 0.90)	0.079
24th month	−5.25 (6.21)	−6.33 (4.89)		
48th month	3.75 (6.28)	−5.50 (4.95)		

PD, Parkinson’s disease; RBD, REM sleep behavior disorder; SD, standard deviation; PDRBD+, RBD-positive PD patients; PDRBD-, RBD-negative PD patients; MDS-UPDRS III, part III of the Movement Disorder Society Unified Parkinson’s Disease Rating Scale; SCOPA-AUT, scales for outcomes in Parkinson’s disease-autonomic; MoCA, Montreal Cognitive Assessment; AI, asymmetry index.

### Longitudinal GMV changes

**Figure 2:**
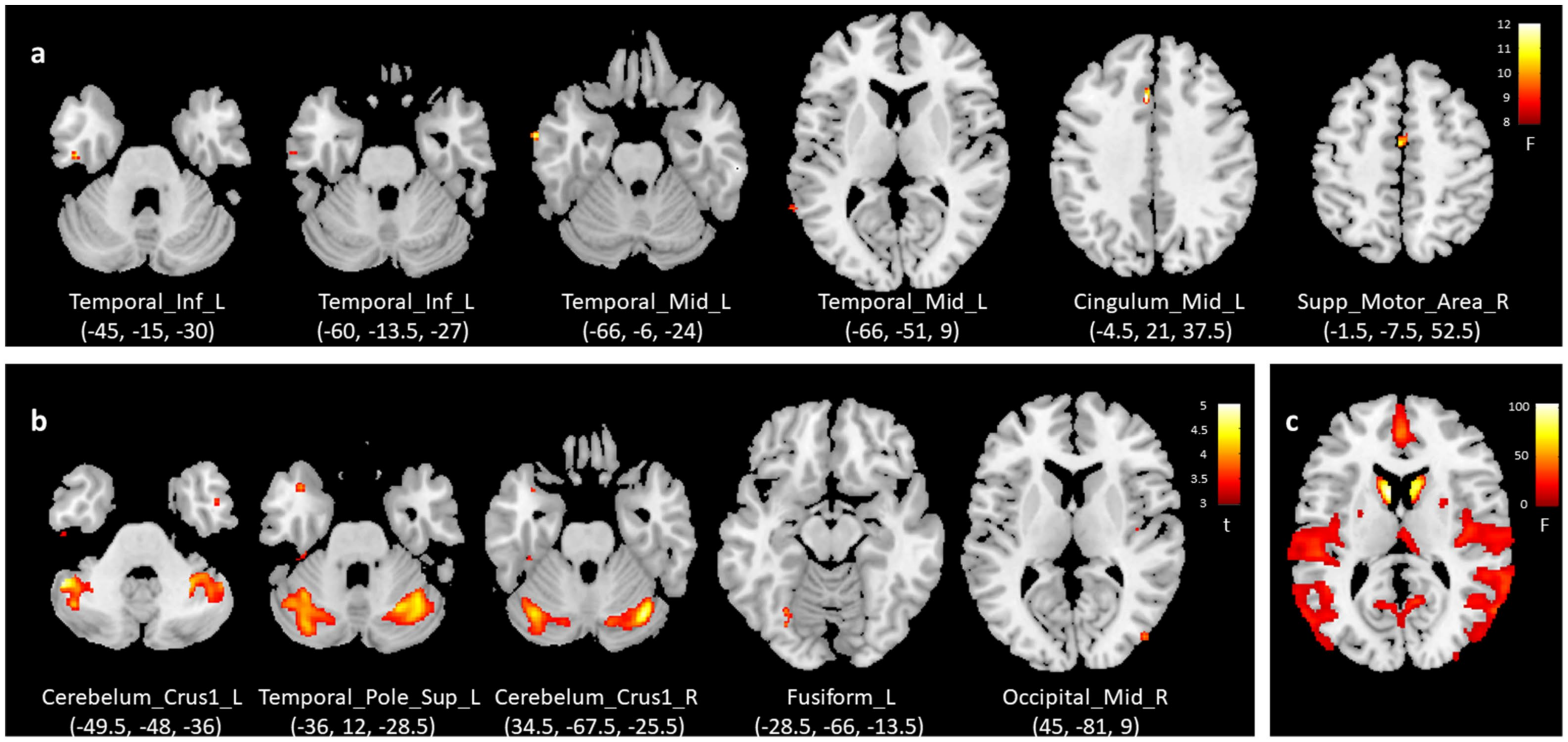
Longitudinal GMV analysis: **(a)** group-by-time interaction, **(b)** comparison between PDRBD+ and PDRBD− groups at 48th month, and **(c)** time effect across all PD patients. GMV, gray matter volume; PD, Parkinson’s disease; RBD, REM sleep behavior disorder; PDRBD+, RBD-positive PD patients; PDRBD−, RBD-negative PD patients, Temporal_Inf_L, left inferior temporal gyrus; Temporal_Mid_L, left middle temporal gyrus; Cingulum_Mid_L, left middle cingulate gyrus; Supp_Motor_Area_R, right supplementary motor area; Cerebelum_Crus1_L, left Crus I of cerebellum; Temporal_Pole_Sup_L, left superior temporal pole; Cerebelum_Crus1_R, Right Crus I of cerebellum; Fusiform_L, left fusiform gyrus; Occipital_Mid_R, right middle occipital gyrus.

Table 3, Figure 2A, and Supplementary Figure 1 showed the group-by-time interaction GMV analysis results and all regions were defined by the Automated Anatomical Labeling (AAL) atlas. Compared with the PDRBD− group, the PDRBD+ group showed GM loss in the mesial surface of frontal lobes and the left temporal cortex (FDR-corrected *p* < 0.05), including left middle cingulum (peak MNI: −4.5, 21, 37.5; η^2^_ₚ_ = 0.19), left middle temporal gyrus (two clusters: peak at −66, −6, −24, η^2^_ₚ_ = 0.18 and −66, −51, 9, η^2^_ₚ_ = 0.16), right supplementary motor area (peak MNI: −1.5, −7.5, 52.5; η^2^_ₚ_ = 0.17), and left inferior temporal gyrus (two clusters: peak at −45, −15, −30, η^2^ₚ = 0.17 and −60, −13.5, −27, η^2^_ₚ_ = 0.15).

**Table 3:** Anatomical locations of significant GMV alterations in the longitudinal analysis.

Anatomical location	*F_3,156_*	Effect size (η^2^_ₚ_)	Cluster size	Peak MNI coordinate		
				x	y	z
Cingulum_Mid_L	12.24	0.19	51	−4.5	21	37.5
Temporal_Mid_L	11.45	0.18	27	−66	−6	−24
Supp_Motor_Area_R	10.87	0.17	64	−1.5	−7.5	52.5
Temporal_Inf_L	10.73	0.17	17	−45	−15	−30
Temporal_Mid_L	9.61	0.16	11	−66	−51	9
Temporal_Inf_L	9.09	0.15	5	−60	−13.5	−27

GMV, gray matter volume; MNI, Montreal Neurological Institute; Temporal_Inf_L, left inferior temporal gyrus; Temporal_Mid_L, left middle temporal gyrus; Cingulum_Mid_L, left middle cingulate gyrus; Supp_Motor_Area_R, right supplementary motor area; L, left; R, right.

Thereafter, in the comparison at each time point, besides the bilateral frontotemporal lobes, the PDRBD+ group showed significant GM atrophy in bilateral Cerebellum Posterior Lobe at 48th month with large effect sizes (FDR-corrected *p* < 0.05, Cohen’s d 1.35 for right Crus I of cerebellum and 1.38 for left Crus I of cerebellum), which did not survive the FDR correction at baseline, 12th and 24th month (individual effect) (Figure 2B; Supplementary Figure 2; Supplementary Table 2). Over time, each PD group showed extensive cortical GM loss and bilateral caudate and putamen atrophy (FDR-corrected *p* < 0.05) (time effect) (Figure 2c). The GM voxel value of the group-by-time interaction effect and two clusters in the cerebellum of the 48th month during follow-up are shown in Supplementary Figures 3a,b.

### Correlation analysis

Pearson’s analysis showed that the voxel value of the region of significant difference in the group-by-time interaction analysis correlated positively with MoCA (*r* = 0.394, *p* = 0.003) and negatively with SCOPA-AUT at the 48th month (*r* = −0.299, *p* = 0.028) (Supplementary Figures 4a,b). However, the altered GMV was not significantly correlated with MDS-UPDRS-III and putamen AI. When conducting the grouping analysis, the voxel value of altered GM was significantly and positively correlated with MoCA at the 48th month in the PDRBD+ group (*r* = 0.564, *p* = 0.002), but no significant correlation was observed in the PDRBD− group (Supplementary Figure 4c). The grouping analysis of other indicators (MDS-UPDRS-III, SCOPA-AUT, and putamen AI) with voxel value of altered GM did not yield any statistically significant results for their correlation.

### Transcriptomic analysis

The Spearman’s correlation analysis between the group-level F-statistic map from the longitudinal analysis and each of the 15,633 individual AHBA genes identified 218 genes (*p* < 0.05, Bonferroni correction) (Supplementary Table 3). To gain insights into the potential biological functions of the genes with expression levels associated with the persumed body-first PD, we performed gene ontology (GO) enrichment analysis on these genes. The correlated genes were enriched in the Pathways of neurodegeneration—multiple diseases, adrenergic signaling in cardiomyocytes and mitogen-activated protein kinase (MAPK) signaling pathway (Supplementary Figure 5). Cell and molecular biology evidence indicated that MAPK signaling underlies neurodegeneration and that *α*-syn appears to be involved in the MAPK pathway (15).

## Discussion

This study examined clinical and GMV changes by comparing PDRBD+ and PDRBD− groups. Our findings revealed that the PDRBD+ group exhibited more pronounced cognitive impairment and autonomic disorders during the follow-up period. Interestingly, at baseline, the PDRBD+ and PDRBD− groups did not demonstrate divergent atrophy patterns in the GMV analysis. However, over time, the PDRBD+ group displayed relatively rapid GMV atrophy in specific frontotemporal regions during the follow-up period (interaction effect), with effect sizes (η^2^_p_ = 0.15–0.19) indicating moderate but consistent neurodegeneration. Furthermore, the altered GMV correlated positively with the MoCA and negatively with the SCOPA-AUT in the 48th month.

The finding that PD patients exhibiting symptoms of RBD demonstrated poorer cognitive performance and autonomic function at follow-up aligns with existing research. Increasing evidence supports the hypothesis that brain-first and body-first PD show different clinical presentations, and PD patients with RBD have more pronounced autonomic dysfunctions and cognitive impairment (16, 17). Conventional clustering analysis often categorizes patients into mild or diffuse-malignant subtypes (18, 19). The diffuse-malignant subtype is characterized by an older age at onset, predominant non-motor manifestations, particularly in the cognitive domain, and a more rapid progression toward significant milestones such as dementia, the need for gait assistance, institutionalization, and mortality (20, 21). In this context, the accelerated deterioration in MoCA scores and worsening SCOPA-AUT scores aligns with the “diffuse-malignant” PD subtype, suggesting RBD status may predict non-motor progression trajectories.

While prior studies primarily focused on the pathological alterations occurring in the brainstem (7, 22, 23), our longitudinal MRI analysis provides the structural evidence comparing whole-brain neurodegeneration patterns between subtypes, revealing selective GM loss in specific frontotemporal regions in PDRBD+. GM loss can be considered a marker of neurodegeneration and, to some extent, suggests the propagation pathway of *α*-syn. α-syn pathology originating in the dorsal locus coeruleus may propagate preferentially via the noradrenergic bundle to neocortex (24, 25), while ventral locus coeruleus involvement could drive cerebellar pathology—a dichotomy recently mapped in human neuropathology (26). This connectivity-driven pattern differs fundamentally from brain-first PD’s olfactory-amygdala origin (6), supporting distinct α-syn spreading mechanisms in PD subtypes. Identification of accelerated frontotemporal atrophy in PDRBD+ patients suggests subtype-specific therapeutic opportunities (e.g., α-syn-targeting antibodies like prasinezumab) and preventive strategies (e.g., lifestyle interventions during the prodromal iRBD-PDRBD+ continuum) for this high-risk population.

Besides the GMV loss in specific frontotemporal regions revealed by group-by-time interaction, PDRBD+ also showed bilateral cerebellum atrophy in the 48th month. The two clusters in the cerebellum were significant at *p* < 0.001 level at each time point, with only the 48th month surviving FDR correction. Additionally, Guo et al. (27) discovered that among PD patients with possible RBD, various cerebellum regions showed significantly diminished nodal properties. In line with this, the iRBD and PD-RBD patients showed consistently reduced blood flow in the cerebellar hemispheres and reduced volume in the cerebellar cortex due to regional neuronal activity impairment (28–30). Thus, our findings are consistent with existing literature indicating the aberrant function and structural changes in the cerebellum in patients with RBD. A recent study has also revealed the presence of *α*-synuclein-related pathological alterations in the cerebellum of individuals with PD. The Lewy bodies identified in the cerebellum in PD patients were speculated to originate from the pre-cerebellar brainstem and propagate in a prion-like manner (31).

Our cross-sectional analysis of gray matter volume did not show diverging atrophy. Despite numerous studies, conflicting evidence exists on GMV atrophy in PD’s early stages, with different patterns reported by MRI studies. These cross-study differences may partly be attributed to substantial methodological heterogeneity. While studies have a consensus regarding the progressive atrophy associated with the disease’s longer duration and higher severity, there is greater variability in establishing the specific time at which atrophy becomes overt (32, 33). These observations align with structural MRI’s recognized detection threshold—its sensitivity to early neurodegeneration is limited by the temporal gap between functional impairment and volumetric loss (34–37) and by resolution constraints for small brainstem nuclei (35).

Surprisingly, the two putative PD subtypes did not significantly differ in putaminal DaT asymmetry at baseline. At the 48th month follow-up, the body-first PD group had significantly higher putamen AI than the brain-first PD group. This result was inconsistent with the previous studies and failed to support the hypothesized symmetric spread of initial enteric pathology to the brainstem, leading to symmetric neurodegeneration in body-first PD (38, 39). While this hypothesis is intriguing, recent cross-sectional studies have found no evidence of either greater DaT uptake asymmetry, interhemispheric differences in PD-related metabolic pattern expression (40), or GMV asymmetry (11). The finding of increased putamen asymmetry in PDRBD+ at 48 months challenges the traditional assumption of symmetric neurodegeneration in body-first PD. While *α*-syn pathology remains central to disease progression, the observed asymmetry may be explained by network-specific vulnerabilities in brainstem-basal ganglia circuits (41), interactions with co-existing pathologies (42), as well as the effects of genetic predisposition (43) and environmental exposures on neuronal vulnerability, which may collectively modify canonical Braak staging patterns in clinical populations (44).

Our study has several limitations. Firstly, PD patients’ RBD status was determined using the RBDSQ score rather than a formal diagnosis by polysomnography. Although we applied more conservative cutoff values, this approach may introduce some uncertainty. Future studies should incorporate more objective measures, such as video-polysomnography, to improve the accuracy of RBD assessment. Additionally, our analysis did not account for potential genetic (e.g., *GBA* or *LRRK2* mutations) or environmental (e.g., smoking) covariates, which may influence disease progression and phenotypic expression. Furthermore, although 48 months of follow-up data were available for two PD subtypes, neither the iRBD nor the HCs group had imaging data for such a long follow-up period. The lack of long-term iRBD and control data is a key limitation, preventing direct comparison of prodromal-to-clinical progression. Specifically, missing iRBD follow-up obscures whether it shares PDRBD+'s neurodegenerative trajectory. Future studies should prioritize longitudinal iRBD monitoring to bridge this gap. Finally, it is worth noting that *α*-syn pathology can only be evaluated by indirect markers instead of being directly measured by imaging, as there is currently no validated *in vivo* method for assessing α-syn deposition.

## Conclusion

Our study generally indicates that relatively rapid GMV loss in specific frontotemporal regions may occur within 6 years of symptom onset in the body-first PD subtype. These results seem consistent with the progressive ascending spread of α-syn to the neocortex in the body-first subtype and support the emerging hypothesis of distinct α-syn spreading patterns.

## Data Availability

The original contributions presented in the study are included in the article/Supplementary material, further inquiries can be directed to the corresponding author.

## Footnotes

^1^ www.ppmi-info.org/access-data-specimens/download-data

^2^ https://www.ppmi-info.org/sites/default/files/docs/002_Protocol_AM4_v3.0_23July2024_Final.pdf

^3^ http://www.ppmi-info.org/

^4^ http://dbm.neuro.uni-jena.de/cat12/

^5^ www.fil.ion.ucl.ac.uk/spm

^6^ http://metascape.org

## Supplementary material

SUPPLEMENTARY FIGURE 1 Multi-planar views of significant gray matter atrophy clusters in PDRBD+ patients. (a–f) Axial (Z), sagittal (X), and coronal (Y) slices showing peak voxels of six significant clusters (FDR-corrected *p* < 0.05). Coordinates in MNI space: **(a)** Temporal_Inf_L (left inferior temporal gyrus; −45, −15, −30); **(b)** Temporal_Inf_L (left inferior temporal gyrus; −60, −13.5, −27); **(c)** Temporal_Mid_L (left middle temporal gyrus; −66, −6, −24); **(d)** Temporal_Mid_L (left middle temporal gyrus; −66, −51, 9); **(e)** Cingulum_Mid_L (left middle cingulate gyrus; −4.5, 21, 37.5); **(f)** Supp_Motor_Area_R (right supplementary motor area; −1.5, −7.5, 52.5).

SUPPLEMENTARY FIGURE 2 Multi-planar views of significant gray matter atrophy clusters between PDRBD+ and PDRBD− patients at 48th month. **(a–e)** Axial (Z), sagittal (X), and coronal (Y) slices showing peak voxels of five significant clusters with size > 30 (FDR-corrected *p* < 0.05). Coordinates in MNI space: **(a)** Cerebelum_Crus1_L (left Crus I of cerebellum; −49.5, −48, −36); **(b)** Temporal_Pole_Sup_L (left superior temporal pole; −36, 12, −28.5); **(c)** Cerebelum_Crus1_R (right Crus I of cerebellum; 34.5, −67.5, −25.5); **(d)** Fusiform_L (left fusiform gyrus; −28.5, −66, −13.5); **(e)** Occipital_Mid_R (right middle occipital gyrus; 45, −81, 9).

SUPPLEMENTARY FIGURE 3 Longitudinal GMV changes between PDRBD+ and PDRBD− groups. **(a)** GMV changes for clusters exhibiting significant group-by-time interaction effects (FDR-corrected *p* < 0.05) across all follow-up visits (baseline, 12th, 24th, and 48th month). **(b)** Longitudinal changes in two cerebellar clusters showing significant individual effect at the 48th month follow-up in post-hoc comparisons. Error bars represent standard errors of the mean. GMV, gray matter volume; PDRBD+, RBD-positive PD patients; PDRBD−, RBD-negative PD patients; FDR, False discovery rate.

SUPPLEMENTARY FIGURE 4 Correlation analysis between altered GMV and clinical measures in PD patients. **(a)** Pearson correlation between voxel values of GMV clusters exhibiting significant group-by-time interaction effects and MoCA at 48^th^ month follow-up for all PD patients. **(b)** Pearson correlation between altered GMV voxel values and SCOPA-AUT at 48th month. **(c)** Group-stratified correlation analysis between altered GMV voxel values and MoCA scores at 48th month time point. GMV, gray matter volume; PDRBD+, RBD-positive PD patients; PDRBD−, RBD-negative PD patients; MoCA, Montreal Cognitive Assessment; SCOPA-AUT, scales for outcomes in Parkinson’s disease-autonomic.

SUPPLEMENTARY FIGURE 5 Enrichment analysis of the correlated genes.
