# Supplementary figures and images for: Rapid frontotemporal gray matter loss in proposed body-first Parkinson’s disease: a longitudinal voxel-based morphometry study
Source: Front Neurol. 2025 Jul 23;16:1579561. doi: 10.3389/fneur.2025.1579561 (PMC12325973; doi:10.3389/fneur.2025.1579561)

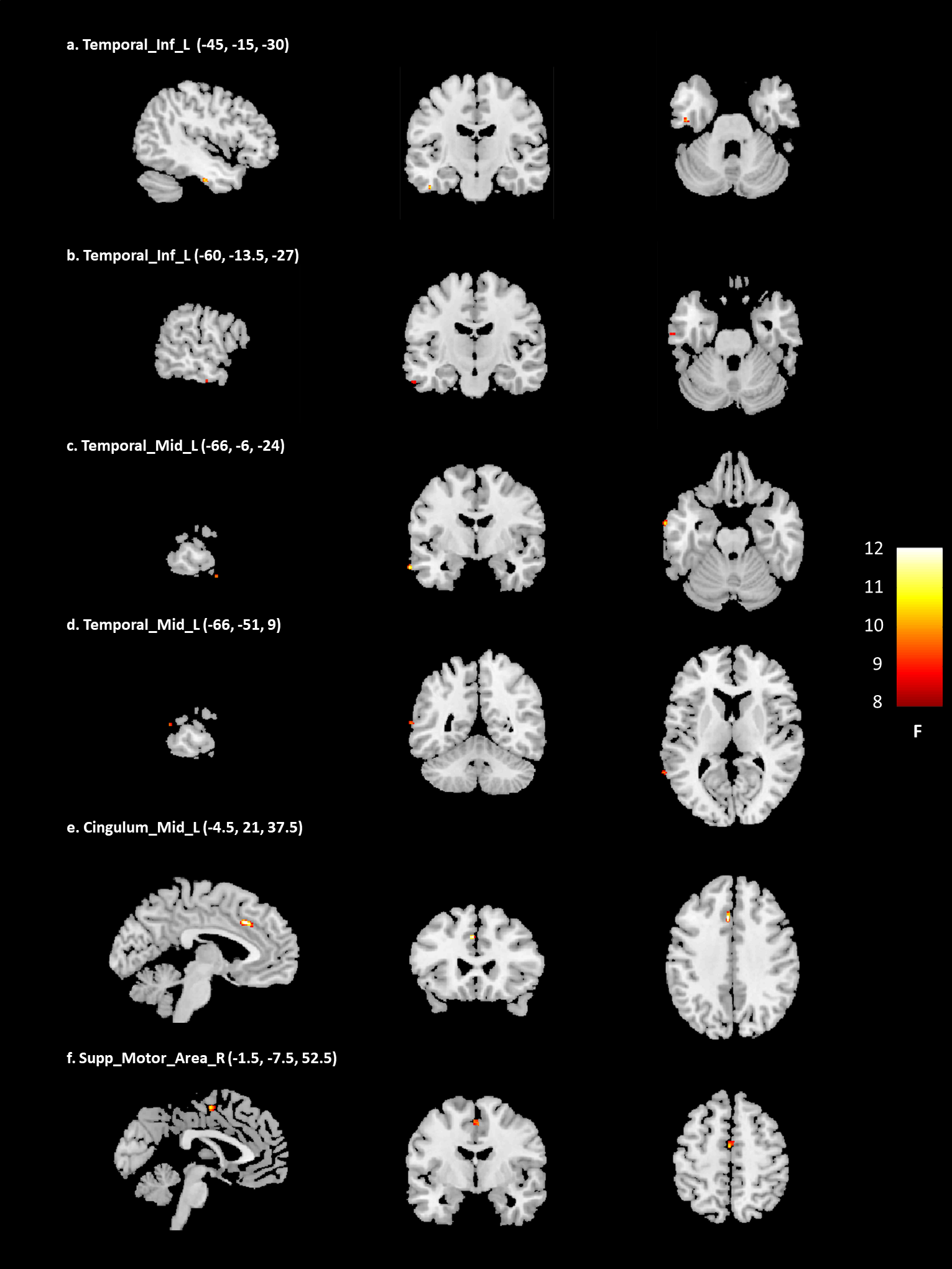

Supplement: SUPPLEMENTARY FIGURE 1 — Multi-planar views of significant gray matter atrophy clusters in PDRBD+ patients. (a–f) Axial (Z), sagittal (X), and coronal (Y) slices showing peak voxels of six significant clusters (FDR-corrected p < 0.05). Coordinates in MNI space: (a) Temporal_Inf_L (left inferior temporal gyrus; −45, −15, −30); (b) Temporal_Inf_L (left inferior temporal gyrus; −60, −13.5, −27); (c) Temporal_Mid_L (left middle temporal gyrus; −66, −6, −24); (d) Temporal_Mid_L (left middle temporal gyrus; −66, −51, 9); (e) Cingulum_Mid_L (left middle cingulate gyrus; −4.5, 21, 37.5); (f) Supp_Motor_Area_R (right supplementary motor area; −1.5, −7.5, 52.5). [file Image_1.TIF]

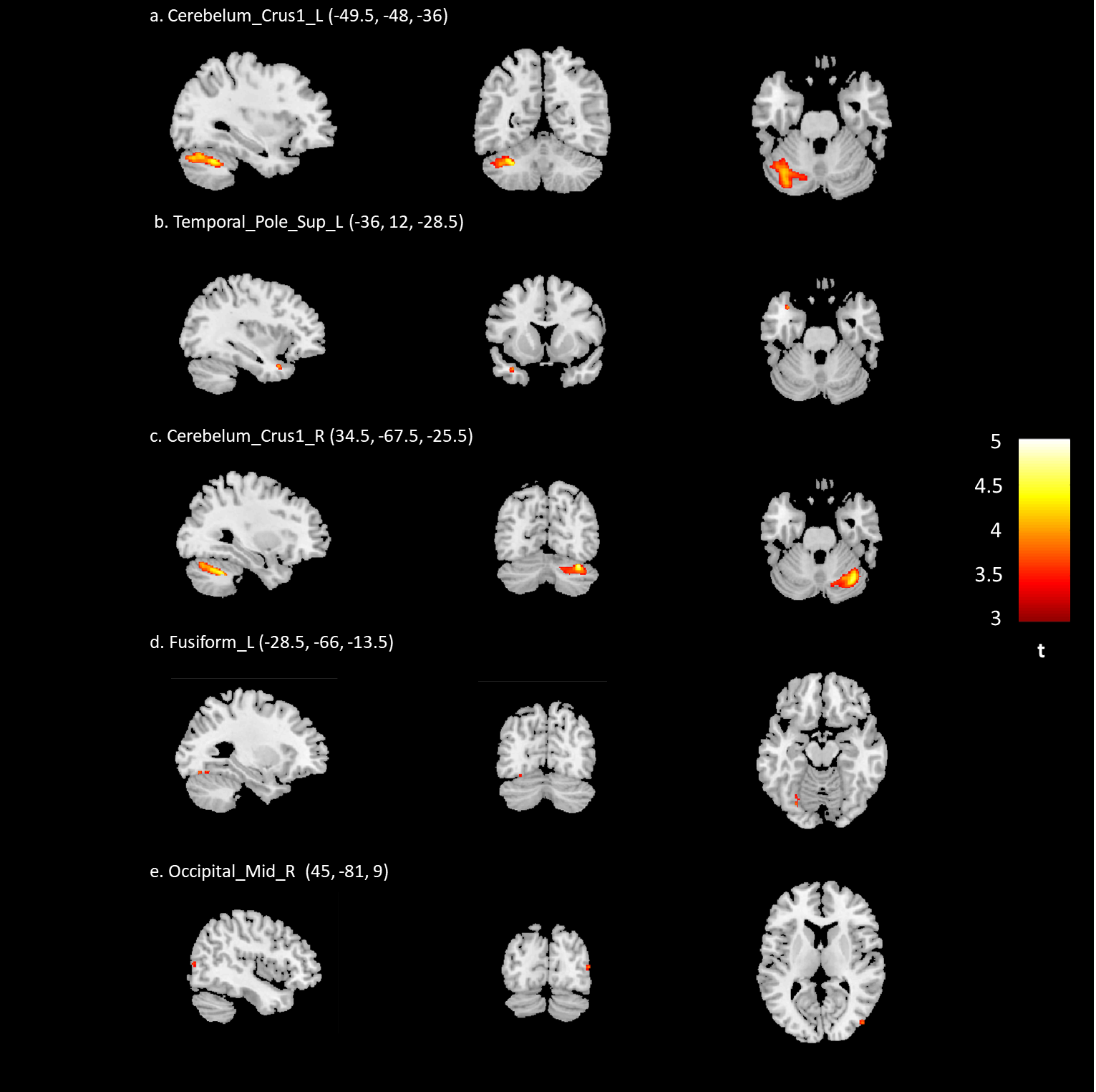

Supplement: SUPPLEMENTARY FIGURE 2 — Multi-planar views of significant gray matter atrophy clusters between PDRBD+ and PDRBD− patients at 48th month. (a–e) Axial (Z), sagittal (X), and coronal (Y) slices showing peak voxels of five significant clusters with size > 30 (FDR-corrected p < 0.05). Coordinates in MNI space: (a) Cerebelum_Crus1_L (left Crus I of cerebellum; −49.5, −48, −36); (b) Temporal_Pole_Sup_L (left superior temporal pole; −36, 12, −28.5); (c) Cerebelum_Crus1_R (right Crus I of cerebellum; 34.5, −67.5, −25.5); (d) Fusiform_L (left fusiform gyrus; −28.5, −66, −13.5); (e) Occipital_Mid_R (right middle occipital gyrus; 45, −81, 9). [file Image_2.TIF]

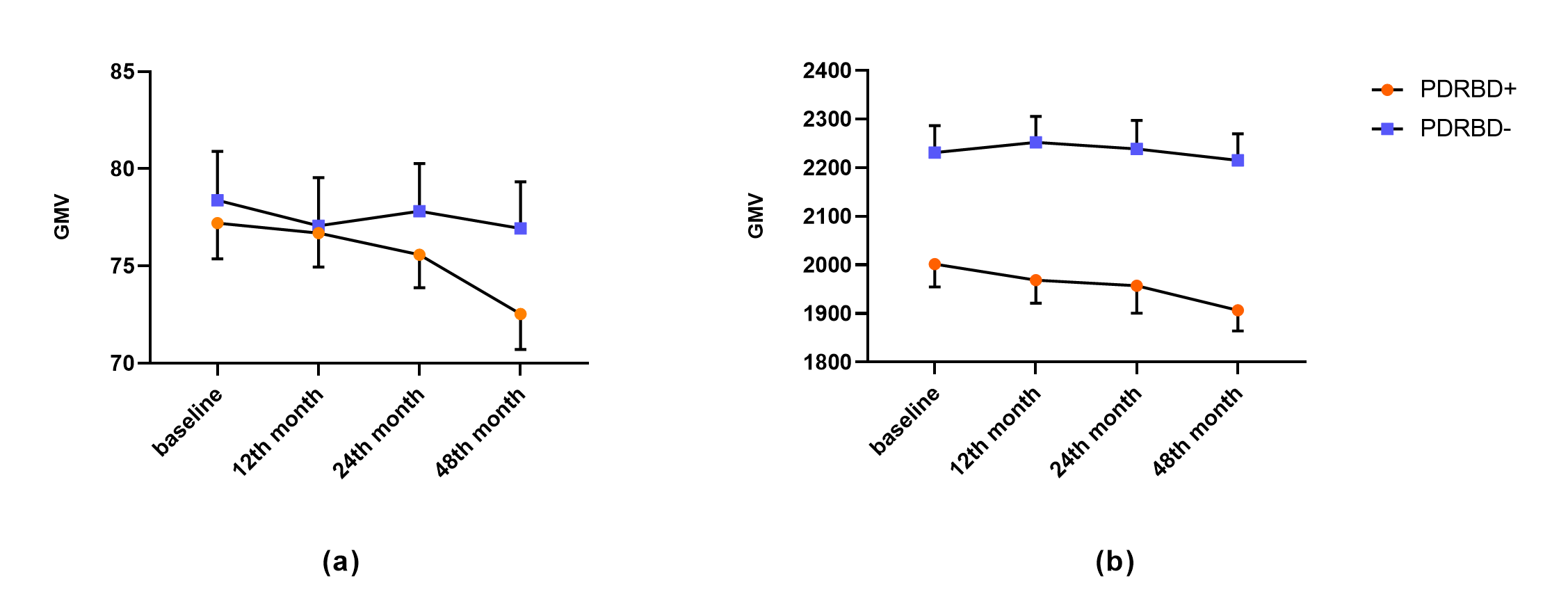

Supplement: SUPPLEMENTARY FIGURE 3 — Longitudinal GMV changes between PDRBD+ and PDRBD− groups. (a) GMV changes for clusters exhibiting significant group-by-time interaction effects (FDR-corrected p < 0.05) across all follow-up visits (baseline, 12th, 24th, and 48th month). (b) Longitudinal changes in two cerebellar clusters showing significant individual effect at the 48th month follow-up in post-hoc comparisons. Error bars represent standard errors of the mean. GMV, gray matter volume; PDRBD+, RBD-positive PD patients; PDRBD−, RBD-negative PD patients; FDR, False discovery rate. [file Image_3.TIF]

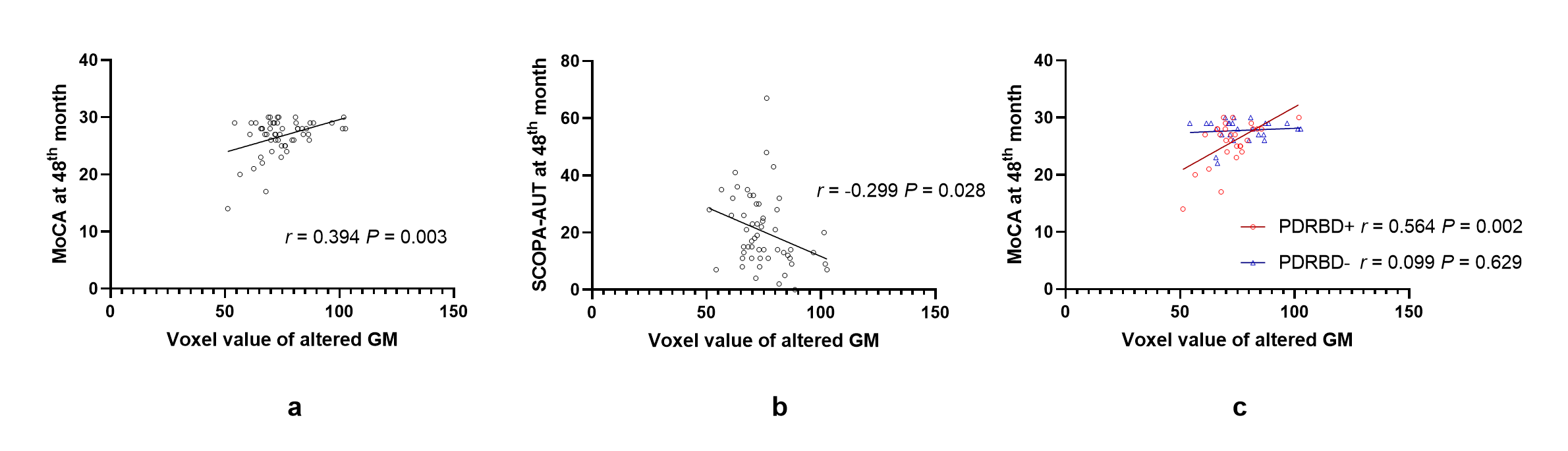

Supplement: SUPPLEMENTARY FIGURE 4 — Correlation analysis between altered GMV and clinical measures in PD patients. (a) Pearson correlation between voxel values of GMV clusters exhibiting significant group-by-time interaction effects and MoCA at 48th month follow-up for all PD patients. (b) Pearson correlation between altered GMV voxel values and SCOPA-AUT at 48th month. (c) Group-stratified correlation analysis between altered GMV voxel values and MoCA scores at 48th month time point. GMV, gray matter volume; PDRBD+, RBD-positive PD patients; PDRBD−, RBD-negative PD patients; MoCA, Montreal Cognitive Assessment; SCOPA-AUT, scales for outcomes in Parkinson’s disease-autonomic. [file Image_4.TIF]

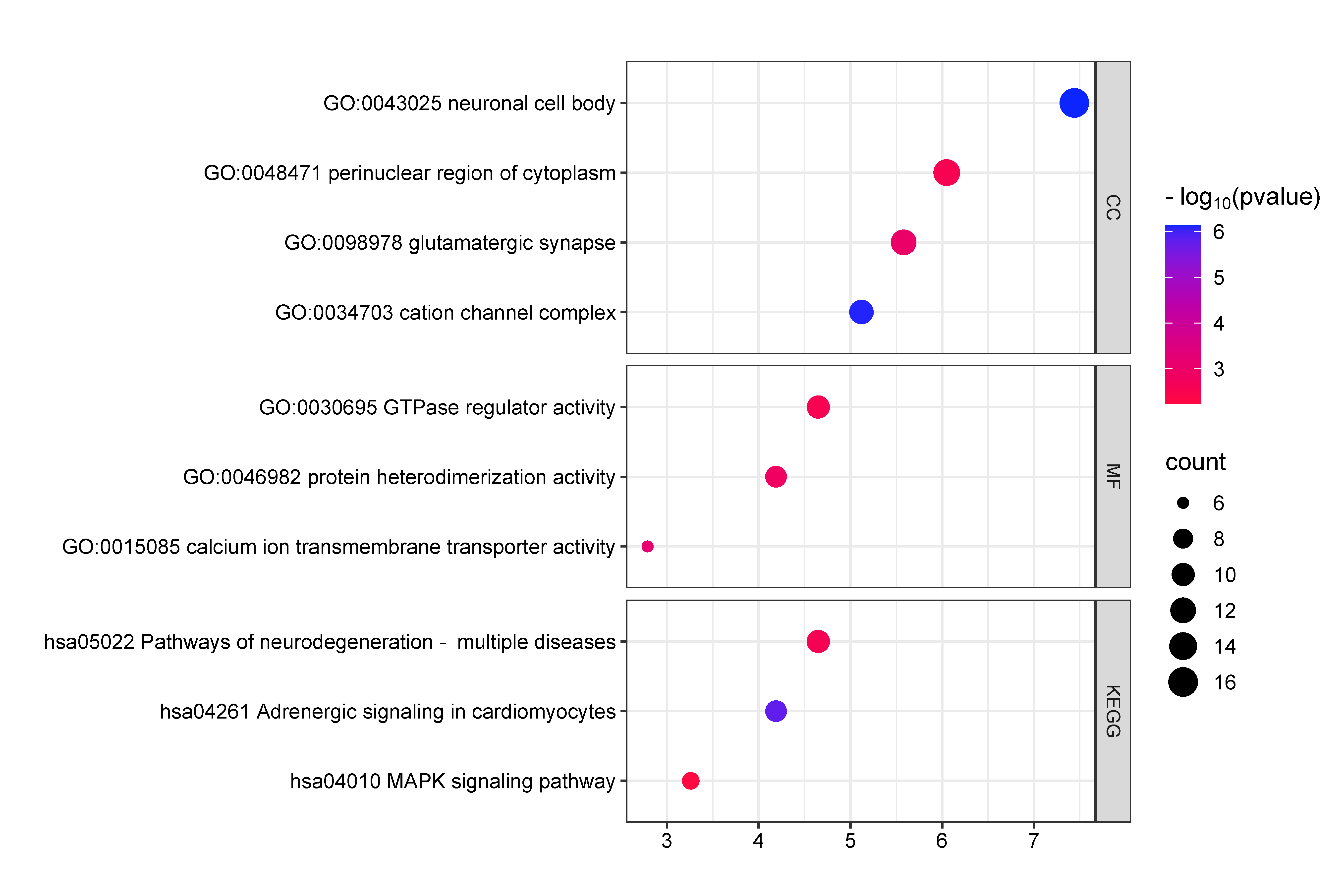

Supplement: SUPPLEMENTARY FIGURE 5 — Enrichment analysis of the correlated genes. [file Image_5.TIFF]
