# Supplementary material for: Rapid frontotemporal gray matter loss in proposed body-first Parkinson’s disease: a longitudinal voxel-based morphometry study
Source: Front Neurol. 2025 Jul 23;16:1579561. doi: 10.3389/fneur.2025.1579561 (PMC12325973; doi:10.3389/fneur.2025.1579561)
Supplement: Supplementary file 6 [file Table_1.docx]

**sTable 1** Raw data during follow-up between PDRBD+ and PDRBD- groups

|  |  | Baseline | 12th month | 24th month | 48th month | *F* | *P* |
| --- | --- | --- | --- | --- | --- | --- | --- |
| MDS-UPDRSIII | | | | | | | |
|  | PDRBD+ | 24.96±10.49 | 23.61±11.84 | 27.96±13.09 | 30.42±13.71 | 6.49 | 0.009 |
|  | PDRBD- | 21.07±7.67 | 21.88±10.44 | 22.48±11.23 | 27.11±8.32 | 4.12 | 0.001 |
|  | *t* | 1.54 | 0.48 | 0.91 | 0.14 |  |  |
|  | *P* | 0.129* | 0.632# | 0.365# | 0.886# |  |  |
| MoCA |  |  |  |  |  |  |  |
|  | PDRBD+ | 26.78±2.48 | 25.43±3.79 | 25.85±3.30 | 25.71±3.83 | 2.00 | 0.120 |
|  | PDRBD- | 27.42±2.02 | 27.38±2.04 | 27.19±2.06 | 27.77±1.97 | 0.63 | 0.596 |
|  | *t* | 1.03 | 2.15 | 1.35 | 2.28 |  |  |
|  | *P* | 0.308* | 0.034# | 0.180# | 0.025# |  |  |
| SCOPA-AUT | | | | | | | |
|  | PDRBD+ | 17.35±13.64 | 18.64±13.76 | 21.86±12.78 | 25.64±13.23 | 9.08 | <0.001 |
|  | PDRBD- | 10.73±8.33 | 12.52±8.73 | 12.84±8.45 | 14.77±9.71 | 4.65 | 0.005 |
|  | *t* | 2.13 | 0.39 | 1.98 | 2.84 |  |  |
|  | *P* | 0.038* | 0.697# | 0.050# | 0.005# |  |  |
| Putamen AI | | | | | | | |
|  | PDRBD+ | 41.68±25.87 | 32.15±21.41 | 34.98±28.76 | 46.16±34.03 | 1.74 | 0.167 |
|  | PDRBD- | 31.10±22.01 | 28.56±18.30 | 24.93±15.26 | 24.50±16.19 | 0.71 | 0.552 |
|  | *t* | 1.57 | 0.07 | 1.18 | 2.41 |  |  |
|  | *P* | 0.122* | 0.944# | 0.242# | 0.017# |  |  |

Abbreviations: PD, Parkinson’s disease; RBD, REM sleep behaviour disorder; PDRBD+, RBD-positive PD patients; PDRBD-, RBD-negative PD patients; MDS‐UPDRS III, part III of the Movement Disorder Society Unified Parkinson's Disease Rating Scale; MoCA, Montreal Cognitive Assessment; SCOPA-AUT, scales for outcomes in Parkinson’s disease-autonomic; AI, asymmetry index.

* by independent *t* test

# by linear mixed-effects model
