# Supplementary material for: Rapid frontotemporal gray matter loss in proposed body-first Parkinson’s disease: a longitudinal voxel-based morphometry study
Source: Front Neurol. 2025 Jul 23;16:1579561. doi: 10.3389/fneur.2025.1579561 (PMC12325973; doi:10.3389/fneur.2025.1579561)
Supplement: Supplementary file 7 [file Table_2.docx]

**sTable 2** Anatomical locations of significant GMV alterations between PDRBD+ and PDRBD- at 48^th^ month

| Anatomical location | *t* | Cohen's d | Cluster size | Peak MNI coordinate | | |
| --- | --- | --- | --- | --- | --- | --- |
|  |  |  |  | x | y | z |
| Cerebelum_Crus1_L | 4.88 | 1.38 | 1667 | -49.5 | -48 | -36 |
| Cerebelum_Crus1_R | 4.76 | 1.35 | 1774 | 34.5 | -67.5 | -25.5 |
| Temporal_Pole_Sup_L | 4.03 | 1.14 | 33 | -36 | 12 | -28.5 |
| Occipital_Mid_R | 3.95 | 1.12 | 29 | 45 | -81 | 9 |
| Supp_Motor_Area_R | 3.85 | 1.09 | 16 | 10.5 | 16.5 | 60 |
| Fusiform_L | 3.83 | 1.08 | 34 | -28.5 | -66 | -13.5 |
| Temporal_Inf_R | 3.66 | 1.04 | 11 | 46.5 | 3 | -36 |
| Temporal_Sup_R | 3.62 | 1.02 | 8 | 60 | -54 | 19.5 |
| Insula_R | 3.60 | 1.02 | 7 | 40.5 | -12 | 7.5 |
| Fusiform_L | 3.59 | 1.02 | 21 | -33 | -28.5 | -30 |
| Lingual_L | 3.53 | 1.00 | 14 | -15 | -51 | -1.5 |
| Temporal_Inf_L | 3.45 | 0.98 | 5 | -49.5 | -18 | -36 |

Abbreviations: GMV, gray matter volume; PDRBD+, RBD-positive PD patients; PDRBD-, RBD-negative PD patients; MNI, Montreal Neurological Institute; Cerebelum_Crus1_L, left crus 1 of cerebellum; Cerebelum_Crus1_R, right crus 1 of cerebellum; Temporal_Pole_Sup_L, left superior temporal pole; Occipital_Mid_R, right middle occipital gyrus; Supp_Motor_Area_R, right supplementary motor area; Fusiform_L, left fusiform gyrus; Temporal_Inf_R, right inferior temporal gyrus; Temporal_Sup_R, right superior temporal gyrus; Insula_R, right insula; Lingual_L, left lingual gyrus; Temporal_Inf_L, left inferior temporal gyrus; L, left; R, right.
