# Supplementary material for: Rapid frontotemporal gray matter loss in proposed body-first Parkinson’s disease: a longitudinal voxel-based morphometry study
Source: Front Neurol. 2025 Jul 23;16:1579561. doi: 10.3389/fneur.2025.1579561 (PMC12325973; doi:10.3389/fneur.2025.1579561)
Supplement: Supplementary file 8 [file Table_3.docx]

**sTable 3** Significantly correlating genes with group-level *F*-statistic map

| Label | Spearman’s correlation coefficients (ρ) | *P*-value | *P*-value Bonferroni-adjusted |
| --- | --- | --- | --- |
| BHLHE40 | 0.620377 | 0 | 0 |
| IL1B | -0.6697 | 0 | 0 |
| PKIB | -0.61947 | 0 | 0 |
| RRP12 | 0.675622 | 0 | 0 |
| SRD5A3 | -0.64649 | 0 | 0 |
| SAMD9 | -0.61626 | 3.73E-11 | 5.83E-07 |
| PTGER4 | -0.6161 | 5.34E-11 | 8.35E-07 |
| SLC39A13 | 0.607489 | 1.25E-09 | 1.95E-05 |
| PRKCB | 0.606755 | 1.40E-09 | 2.19E-05 |
| TRPM2 | 0.60136 | 2.73E-09 | 4.27E-05 |
| CPLX1 | 0.593531 | 5.83E-09 | 9.11E-05 |
| LPCAT4 | 0.588766 | 8.70E-09 | 0.000136 |
| GASAL1 | -0.58639 | 1.05E-08 | 0.000164 |
| GUCY1A2 | 0.583162 | 1.34E-08 | 0.000209 |
| FAM78A | 0.572898 | 2.80E-08 | 0.000438 |
| PLAGL1 | 0.571974 | 2.99E-08 | 0.000467 |
| GHRLOS | 0.570673 | 3.26E-08 | 0.00051 |
| GPR158 | 0.570589 | 3.28E-08 | 0.000513 |
| TLL1 | -0.56901 | 3.65E-08 | 0.000571 |
| CIT | 0.566916 | 4.19E-08 | 0.000655 |
| RNASEH2A | -0.56654 | 4.30E-08 | 0.000672 |
| SERTAD4 | 0.566412 | 4.33E-08 | 0.000677 |
| SCN4B | 0.566118 | 4.42E-08 | 0.000691 |
| GPC4 | -0.56536 | 4.64E-08 | 0.000725 |
| PLEKHA6 | 0.564754 | 4.83E-08 | 0.000755 |
| IFI27L1 | -0.56324 | 5.33E-08 | 0.000833 |
| ATXN1 | 0.562193 | 5.71E-08 | 0.000893 |
| REPIN1 | 0.561899 | 5.82E-08 | 0.00091 |
| FER1L4 | 0.560892 | 6.21E-08 | 0.000971 |
| FZD4 | 0.560136 | 6.51E-08 | 0.001018 |
| ZNF106 | 0.558919 | 7.04E-08 | 0.001101 |
| SLC24A2 | 0.558877 | 7.06E-08 | 0.001104 |
| MIR29B2CHG | 0.557701 | 7.60E-08 | 0.001188 |
| SLC25A37 | 0.557701 | 7.60E-08 | 0.001188 |
| PRAG1 | 0.557071 | 7.91E-08 | 0.001237 |
| MATN2 | -0.55619 | 8.36E-08 | 0.001307 |
| RILP | 0.555959 | 8.48E-08 | 0.001326 |
| LMLN | 0.554931 | 9.05E-08 | 0.001415 |
| ANKS1A | 0.554637 | 9.22E-08 | 0.001441 |
| PLCB1 | 0.55449 | 9.30E-08 | 0.001454 |
| LMO1 | -0.55384 | 9.69E-08 | 0.001515 |
| C1RL | 0.553293 | 1.00E-07 | 0.001563 |
| SCRT1 | 0.552643 | 1.04E-07 | 0.001626 |
| RGS18 | -0.55252 | 1.05E-07 | 0.001641 |
| LINC00260 | -0.55132 | 1.13E-07 | 0.001767 |
| ATP10A | 0.550229 | 1.21E-07 | 0.001892 |
| STAC2 | 0.550187 | 1.21E-07 | 0.001892 |
| ZNF727 | -0.55 | 1.23E-07 | 0.001923 |
| IMPDH1 | 0.549746 | 1.25E-07 | 0.001954 |
| ZCCHC12 | -0.54773 | 1.41E-07 | 0.002204 |
| LRPAP1 | 0.547458 | 1.43E-07 | 0.002236 |
| ARHGEF17 | 0.546891 | 1.48E-07 | 0.002314 |
| KNG1 | 0.546283 | 1.54E-07 | 0.002407 |
| ECHDC3 | -0.54616 | 1.55E-07 | 0.002423 |
| CACNB4 | 0.545968 | 1.57E-07 | 0.002454 |
| KIF2A | -0.54584 | 1.58E-07 | 0.00247 |
| TIMP2 | -0.54477 | 1.69E-07 | 0.002642 |
| FAM105A | -0.54282 | 1.90E-07 | 0.00297 |
| DNAJC4 | 0.542442 | 1.94E-07 | 0.003033 |
| SEMA3D | -0.54122 | 2.08E-07 | 0.003252 |
| PPARGC1A | 0.540888 | 2.13E-07 | 0.00333 |
| ISG15 | -0.54068 | 2.15E-07 | 0.003361 |
| NR2F2 | -0.54068 | 2.15E-07 | 0.003361 |
| LZTS3 | 0.54007 | 2.23E-07 | 0.003486 |
| SLC5A6 | 0.539944 | 2.25E-07 | 0.003517 |
| CPNE6 | -0.53946 | 2.31E-07 | 0.003611 |
| SLA | -0.53948 | 2.31E-07 | 0.003611 |
| RAD54B | 0.539146 | 2.36E-07 | 0.003689 |
| DR1 | -0.53887 | 2.40E-07 | 0.003752 |
| ARHGAP6 | -0.53845 | 2.46E-07 | 0.003846 |
| ZCCHC18 | -0.53808 | 2.51E-07 | 0.003924 |
| ATG16L1 | 0.536354 | 2.78E-07 | 0.004346 |
| SLC9A1 | 0.536354 | 2.78E-07 | 0.004346 |
| ZMAT4 | 0.536333 | 2.78E-07 | 0.004346 |
| TMEM86B | 0.533458 | 3.29E-07 | 0.005143 |
| PRKG1 | -0.53342 | 3.30E-07 | 0.005159 |
| BAK1 | 0.532387 | 3.50E-07 | 0.005472 |
| INTS4P1 | 0.53138 | 3.71E-07 | 0.0058 |
| PCSK1 | 0.530666 | 3.86E-07 | 0.006034 |
| ARHGAP36 | -0.53062 | 3.87E-07 | 0.00605 |
| GEMIN8P4 | -0.53048 | 3.91E-07 | 0.006113 |
| FOSB | 0.530435 | 3.92E-07 | 0.006128 |
| PDE4A | 0.530058 | 4.00E-07 | 0.006253 |
| PFKFB2 | 0.529302 | 4.18E-07 | 0.006535 |
| SPATA33 | -0.52926 | 4.19E-07 | 0.00655 |
| CD99L2 | 0.529029 | 4.25E-07 | 0.006644 |
| SLCO4A1 | 0.528001 | 4.50E-07 | 0.007035 |
| PCDH18 | -0.52794 | 4.52E-07 | 0.007066 |
| KCNA1 | 0.527266 | 4.69E-07 | 0.007332 |
| ACTN4 | 0.526384 | 4.94E-07 | 0.007723 |
| HCST | -0.52624 | 4.98E-07 | 0.007785 |
| CLEC16A | 0.525713 | 5.13E-07 | 0.00802 |
| APBB1IP | -0.52567 | 5.14E-07 | 0.008035 |
| P2RY12 | -0.52527 | 5.26E-07 | 0.008223 |
| SULF2 | -0.52504 | 5.33E-07 | 0.008332 |
| NAT8L | 0.524684 | 5.43E-07 | 0.008489 |
| ZNF677 | -0.52466 | 5.44E-07 | 0.008504 |
| POU6F1 | 0.524621 | 5.45E-07 | 0.00852 |
| FAM181A | -0.52437 | 5.53E-07 | 0.008645 |
| TAPBP | 0.52418 | 5.59E-07 | 0.008739 |
| PLXDC1 | 0.524117 | 5.61E-07 | 0.00877 |
| RNASEH2C | -0.52376 | 5.72E-07 | 0.008942 |
| CD84 | -0.52334 | 5.86E-07 | 0.009161 |
| CLEC2L | 0.523194 | 5.91E-07 | 0.009239 |
| CCIN | -0.52256 | 6.12E-07 | 0.009567 |
| HNRNPUL2 | 0.522396 | 6.18E-07 | 0.009661 |
| EEPD1 | 0.522375 | 6.19E-07 | 0.009677 |
| SLC16A8 | -0.52231 | 6.21E-07 | 0.009708 |
| CNNM1 | 0.521955 | 6.34E-07 | 0.009911 |
| KCTD4 | -0.52175 | 6.41E-07 | 0.010021 |
| PYDC1 | -0.52162 | 6.46E-07 | 0.010099 |
| FABP7 | -0.5212 | 6.61E-07 | 0.010333 |
| PCP4 | 0.520612 | 6.83E-07 | 0.010677 |
| PCNT | 0.52057 | 6.85E-07 | 0.010709 |
| UBXN8 | -0.52055 | 6.85E-07 | 0.010709 |
| LRRC37A4P | 0.520381 | 6.92E-07 | 0.010818 |
| XKR6 | 0.520108 | 7.02E-07 | 0.010974 |
| TFEC | -0.52009 | 7.03E-07 | 0.01099 |
| NCEH1 | 0.519814 | 7.14E-07 | 0.011162 |
| ETV6 | 0.519793 | 7.15E-07 | 0.011178 |
| CAPZB | -0.51965 | 7.21E-07 | 0.011271 |
| KCNQ5 | 0.51908 | 7.44E-07 | 0.011631 |
| PCDHB16 | 0.518996 | 7.47E-07 | 0.011678 |
| TTLL11 | 0.518366 | 7.74E-07 | 0.0121 |
| PPCS | -0.51835 | 7.75E-07 | 0.012116 |
| PPP1CB | 0.518051 | 7.88E-07 | 0.012319 |
| LOC102724156 | -0.51704 | 8.33E-07 | 0.013022 |
| TRPC3 | 0.516729 | 8.47E-07 | 0.013241 |
| ANKRD18DP | 0.51612 | 8.76E-07 | 0.013695 |
| OSBPL3 | 0.51612 | 8.76E-07 | 0.013695 |
| RRS1 | 0.515826 | 8.90E-07 | 0.013913 |
| C12orf75 | -0.51551 | 9.06E-07 | 0.014163 |
| KCNB1 | 0.515071 | 9.28E-07 | 0.014507 |
| TNNI3 | -0.51476 | 9.44E-07 | 0.014758 |
| SCPEP1 | -0.51417 | 9.75E-07 | 0.015242 |
| ZCCHC17 | -0.51415 | 9.76E-07 | 0.015258 |
| MYC | 0.513853 | 9.92E-07 | 0.015508 |
| PIM3 | 0.513664 | 1.00E-06 | 0.015633 |
| GHR | -0.51358 | 1.01E-06 | 0.015789 |
| GTF2IRD2 | -0.51354 | 1.01E-06 | 0.015789 |
| PCDHB17P | 0.513517 | 1.01E-06 | 0.015789 |
| SLC35A4 | 0.513287 | 1.02E-06 | 0.015946 |
| HR | 0.512972 | 1.04E-06 | 0.016258 |
| FBXO33 | 0.51251 | 1.07E-06 | 0.016727 |
| ANKRD37 | 0.511922 | 1.10E-06 | 0.017196 |
| CDC42 | -0.5119 | 1.10E-06 | 0.017196 |
| NDUFB1 | -0.51157 | 1.12E-06 | 0.017509 |
| DND1 | 0.511439 | 1.13E-06 | 0.017665 |
| TBC1D16 | 0.511481 | 1.13E-06 | 0.017665 |
| LINC01128 | 0.511209 | 1.15E-06 | 0.017978 |
| SFXN1 | 0.510789 | 1.17E-06 | 0.018291 |
| ZNF787 | 0.510243 | 1.21E-06 | 0.018916 |
| P2RX6 | 0.509844 | 1.23E-06 | 0.019229 |
| EIF5 | 0.509697 | 1.24E-06 | 0.019385 |
| LINC01102 | 0.509571 | 1.25E-06 | 0.019541 |
| POU3F1 | 0.509089 | 1.29E-06 | 0.020167 |
| NDUFS1 | 0.508753 | 1.31E-06 | 0.020479 |
| ACVR1C | 0.508165 | 1.35E-06 | 0.021105 |
| VBP1 | -0.508 | 1.36E-06 | 0.021261 |
| BLVRB | -0.5077 | 1.39E-06 | 0.02173 |
| EPHB6 | 0.50764 | 1.39E-06 | 0.02173 |
| NR3C1 | 0.507367 | 1.41E-06 | 0.022043 |
| GPR63 | 0.506969 | 1.44E-06 | 0.022512 |
| ANKH | 0.506528 | 1.48E-06 | 0.023137 |
| KCNA3 | 0.505352 | 1.57E-06 | 0.024544 |
| CENPW | -0.50491 | 1.61E-06 | 0.025169 |
| SCN9A | -0.50443 | 1.65E-06 | 0.025794 |
| PNKD | 0.504219 | 1.67E-06 | 0.026107 |
| NFIC | 0.503694 | 1.72E-06 | 0.026889 |
| ESRRA | 0.503023 | 1.78E-06 | 0.027827 |
| ATP2B2 | 0.502918 | 1.79E-06 | 0.027983 |
| LINC01158 | 0.502834 | 1.80E-06 | 0.028139 |
| ADAM23 | 0.502477 | 1.83E-06 | 0.028608 |
| HIST1H2BK | -0.50218 | 1.86E-06 | 0.029077 |
| SOHLH1 | 0.502141 | 1.86E-06 | 0.029077 |
| MIR600HG | 0.501868 | 1.89E-06 | 0.029546 |
| OGDHL | 0.501826 | 1.90E-06 | 0.029703 |
| SCN1B | 0.501196 | 1.96E-06 | 0.030641 |
| ATP13A3 | 0.501091 | 1.97E-06 | 0.030797 |
| DEXI | 0.500819 | 2.00E-06 | 0.031266 |
| PTH2 | -0.50082 | 2.00E-06 | 0.031266 |
| KCNT1 | 0.500714 | 2.01E-06 | 0.031422 |
| PCDHB9 | 0.500756 | 2.01E-06 | 0.031422 |
| SLC38A1 | 0.500651 | 2.02E-06 | 0.031579 |
| EXTL2 | 0.50042 | 2.04E-06 | 0.031891 |
| SCN1A | 0.500042 | 2.08E-06 | 0.032517 |
| CTIF | 0.499937 | 2.09E-06 | 0.032673 |
| SLC7A8 | 0.499958 | 2.09E-06 | 0.032673 |
| ZBTB21 | 0.49937 | 2.16E-06 | 0.033767 |
| EFNA5 | 0.498657 | 2.24E-06 | 0.035018 |
| PPP5D1 | -0.49868 | 2.24E-06 | 0.035018 |
| CDC42P3 | -0.49805 | 2.31E-06 | 0.036112 |
| GPCPD1 | 0.498006 | 2.32E-06 | 0.036269 |
| SYT12 | 0.498006 | 2.32E-06 | 0.036269 |
| DHRS2 | -0.4975 | 2.38E-06 | 0.037207 |
| HMCES | 0.496873 | 2.46E-06 | 0.038457 |
| CCDC189 | -0.49664 | 2.49E-06 | 0.038926 |
| CORO6 | 0.496642 | 2.49E-06 | 0.038926 |
| NABP1 | -0.49649 | 2.51E-06 | 0.039239 |
| SGIP1 | 0.496453 | 2.51E-06 | 0.039239 |
| SMIM14 | -0.49631 | 2.53E-06 | 0.039551 |
| NBDY | -0.4962 | 2.55E-06 | 0.039864 |
| SORL1 | 0.496033 | 2.57E-06 | 0.040177 |
| CACNA2D3 | 0.49576 | 2.60E-06 | 0.040646 |
| ARHGAP18 | -0.49561 | 2.62E-06 | 0.040958 |
| SNCG | -0.4954 | 2.65E-06 | 0.041427 |
| SEC14L5 | 0.495193 | 2.68E-06 | 0.041896 |
| MPP1 | 0.495151 | 2.69E-06 | 0.042053 |
| MRPS6 | -0.49505 | 2.70E-06 | 0.042209 |
| PSMB2 | -0.4949 | 2.72E-06 | 0.042522 |
| FGD5 | 0.494354 | 2.80E-06 | 0.043772 |
| OSBP2 | 0.493871 | 2.87E-06 | 0.044867 |
| TCHH | 0.493913 | 2.87E-06 | 0.044867 |
| MNDA | -0.49257 | 3.07E-06 | 0.047993 |
| IER2 | 0.492381 | 3.10E-06 | 0.048462 |
| NKX1-2 | 0.492318 | 3.11E-06 | 0.048619 |
| MX1 | 0.492129 | 3.14E-06 | 0.049088 |
| PLEKHH3 | 0.491919 | 3.18E-06 | 0.049713 |
